# Three-dimensional and nanoscale resolved hierarchical structure of electroplated zinc complex in aqueous zinc battery

**DOI:** 10.1093/nsr/nwag114

**Published:** 2026-04-13

**Authors:** Junyan Li, Xun Guan, Jing Wang, Ge Zhang, John Holoubek, Yi Cui, Yuqi Li, Haoya Wang, Wah Chiu, Yi Cui

**Affiliations:** Department of Materials Science and Engineering, Stanford University, Stanford, CA 94305, USA; Department of Materials Science and Engineering, Stanford University, Stanford, CA 94305, USA; Department of Materials Science and Engineering, Stanford University, Stanford, CA 94305, USA; Department of Materials Science and Engineering, Stanford University, Stanford, CA 94305, USA; Department of Materials Science and Engineering, Stanford University, Stanford, CA 94305, USA; Department of Materials Science and Engineering, Stanford University, Stanford, CA 94305, USA; Department of Materials Science and Engineering, Stanford University, Stanford, CA 94305, USA; Department of Materials Science and Engineering, Stanford University, Stanford, CA 94305, USA; Department of Bioengineering, Stanford University, Stanford, CA 94305, USA; Department of Microbiology and Immunology, Stanford University School of Medicine, Stanford, CA 94305, USA; Division of CryoEM and Bioimaging, SSRL, SLAC National Accelerator Laboratory, Menlo Park, CA 94025, USA; Department of Materials Science and Engineering, Stanford University, Stanford, CA 94305, USA; Stanford Institute for Materials and Energy Sciences, SLAC National Accelerator Laboratory, Menlo Park, CA 94025, USA; Department of Energy Science and Engineering, Stanford University, Stanford, CA 94305, USA

**Keywords:** aqueous zinc battery, transmission electron microscopy, cryogenic electron tomography

## Abstract

Aqueous zinc batteries offer safety and cost-effectiveness for grid-scale energy storage although the electrochemical and chemical corrosion of zinc in water results in complex Zn species and 3D morphology, ultimately degrading battery performance. Thus far, the atomic and nanoscale 3D structure of the electroplated Zn complex remains unclear. Here, by employing advanced transmission electron microscopy, particularly cryogenic electron tomography, we resolve the preserved 3D architecture of electroplated zinc. A hierarchical solid–electrolyte interphase (SEI) comprising two critical structures that could impact battery performance is delineated—an epitaxial ZnO nanolayer on a Zn nanoplate as the inner SEI and petal-like zinc hydroxide sulfate (ZHS) flakes emerging from the edges of a Zn–ZnO crystal as the extended SEI. We discovered three epitaxial conditions of ZnO on electrochemically plated Zn nanocrystals: (0001)_ZnO_ ∥ (0001)_Zn_, (10$\overline{1}$0)_ZnO_ ∥ (10$\overline{1}$0)_Zn_ and (0001)_ZnO_ ∥ (10$\overline{1}$0)_Zn_. This complex Zn–ZnO–ZHS structure implies a correlation between the zinc-crystal edges and the heterogeneous chemical environment, which can be correlated with the zinc-texture- dependent battery performance.

## Teaser

The three-dimensional nanoscale architecture of electroplated zinc is resolved by advanced TEM and cryoET, revealing the spatially heterogeneous electrochemical reactivity and informing the design of high-performance aqueous zinc batteries.

## INTRODUCTION

**Figure 1.**
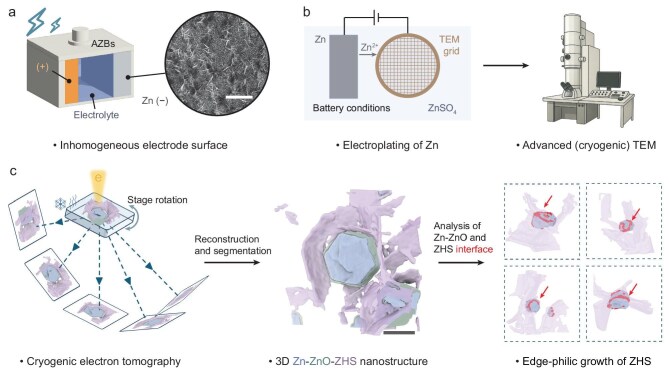
Schematic illustration of electroplated zinc complexes in AZBs. (a) Conventional AZB for grid-scale energy storage. The inset scanning-electron microscopy image shows the inhomogeneous electroplating of zinc. (b) Schematic of zinc electroplating in Zn–Cu half cells used for advanced (cryogenic) transmission electron microscopy (TEM) studies. The instrument artwork on the right was created with assistance from ChatGPT (OpenAI). (c) Schematic of cryogenic electron tomography (cryoET) data collection (left), 3D nanostructure of Zn–ZnO–ZHS complex reconstructed from CryoET data (middle) and the origin of ZHS growth derived from interface analysis of cryoET data (right). Scale bars correspond to 5 μm in (a) and 100 nm in (c), respectively.

Rechargeable aqueous zinc batteries (AZBs) have emerged as a promising candidate for large-scale energy storage [1,2]. On the zinc negative electrodes of these batteries, reversible redox reactions between the zinc ions and zinc metal (Zn^2+^ + 2*e*^−^ = Zn, *E*^o^ = −0.76 V vs. standard hydrogen electrode (SHE)) occur in a water-based electrolyte, offering a comparable energy density (820 mAh·g^−1^ or 5855 mAh·cm^−3^) to those of conventional lithium batteries [3–5]. The water-based electrolyte provides safety, sustainability and cost-effectiveness for battery operations. However, water-related chemical reactions—including the thermodynamically more favorable hydrogen-evolution reaction (HER, 2H^+^ + 2*e*^−^ = H_2_, *E*^o^ = 0.00 V vs. SHE) and the spontaneous corrosion of zinc by water molecules—can cause liquid turbulence and morphology disturbance due to the H_2_ bubble generation at the surface of the zinc electrode [6,7]. Moreover, these interfacial reactions accumulate hydroxide ions (OH^−^) locally, which induces the formation of undesirable by-products at the electrode surface, such as zinc hydroxide sulfate salt (ZHS) in the commonly used zinc sulfate (ZnSO_4_) electrolyte. These instability issues of water significantly unhomogenize the electrode surface and hamper the normal operation of the zinc negative electrodes during battery use (Fig. 1a), leading to reduced coulombic efficiency and shortened lifetime. Therefore, understanding the interfacial structure and chemistry of zinc negative electrodes at the nanoscale is essential for the rational design of long-life AZBs [8].

Typically, obtaining structural and compositional information on zinc negative electrodes relies on the use of ensemble averaging techniques—such as X-ray diffraction or spectroscopies—and imaging techniques with limited spatial resolutions, such as optical or scanning-electron microscopy [9–11]. While these methods offer valuable information on the bulk properties of the materials, they are greatly limited at resolving the spatial heterogeneity of the properties of the materials near the solid–liquid interface, where key electrochemical and chemical reactions occur. Transmission electron microscopy (TEM), on the other hand, provides spatially resolved details of the atomic arrangement, crystal phases and chemical signatures at the nanoscale, enabling the study of structural heterogeneities with appreciable spatial resolution [12–16]. However, conventional TEM suffers from two major limitations: (i) the high-energy electron beam can disrupt beam-sensitive materials, especially those with structural water [17,18], and (ii) the inherently 2D projection hinders accurate visualization of the complex 3D architecture [19–21]. Notably, ultra-low-dose TEM at ambient temperatures has recently demonstrated that atomic-resolution imaging and the diffraction of beam-sensitive battery materials can be achieved, providing complementary routes to mitigate beam-induced artifacts [22,23].

Recent advances in cryogenic TEM (cryoTEM) have revolutionized the characterization and understanding of solid–liquid interfaces in electrochemical systems, especially in energy-storage systems [24,25]. The cryogenic treatment can preserve the metastable materials, thereby allowing the discovery of features that were previously hidden under conventional TEM. For instance, cryoTEM has facilitated the discovery of previously unobservable features in lithium-based batteries, including the discovery of the metastable LiH and the dynamic swelling of the solid–electrolyte interphase (SEI) [26,27]. More notably, the integration of cryoTEM with electron tomography—cryogenic electron tomography (cryoET)—extends the conventional 2D imaging and allows the 3D reconstruction of complex material architectures while maintaining structural integrity under electron irradiation [28–31]. These capabilities are especially well suited for examining the inhomogeneous, multiscale and beam-sensitive compositions of zinc negative electrodes.

Here, we applied TEM and cryoET techniques to investigate the complex structure of electroplated zinc on the zinc negative electrode under battery-relevant conditions (Fig. 1b and c), aiming to elucidate the material structures at and near the electrode surface and to correlate these findings with the chemical gradients around the electrochemical double layer. We discovered an SEI on electroplated zinc metals comprising: (i) a thin passivation layer of ZnO coating on the zinc metal surface, similar to the common SEIs in other battery systems, and (ii) petal-like extended ZHS flakes predominantly emanating from the edges of the Zn–ZnO complex (Fig. 1c). We hypothesize that this extended SEI structure reflects the non-uniform distribution of ions within and near the double-layer region, particularly the OH^−^ ions, with the crystal edges possibly serving as preferential sites for OH^−^ generation and ZHS nucleation. Such a correlation aligns with reported observations that battery performance is affected by the zinc textures.

## RESULTS

### Preparation of electroplated zinc under battery-relevant conditions

The zinc samples were prepared by electroplating in a 2 M ZnSO_4_ electrolyte (pH = 3.9∼4.5), mimicking the conditions of a zinc–copper half-cell. A TEM grid coated with a single layer of graphene and a piece of fresh zinc foil were positioned next to each other—deploying as the working and counter electrodes, respectively—to facilitate crystal growth on the open areas of the TEM grids (Fig. 1b, and see Methods section in the Supplementary data for details). The use of single-layer graphene, which is electron-transparent, enables the controlled growth of well-crystallized zinc crystals [32,33], making it an ideal substrate for studying the detailed structure of electroplated zinc. Typically, zinc crystals were deposited by applying a constant current of −10 mA to the TEM grid for a short duration, ensuring not to exceed the mass transfer limit of zinc ions [34]. Here, we use the term ‘battery-relevant’ to refer specifically to using the common ZnSO_4_ electrolyte and probing the Zn–electrolyte interfacial chemistry during galvanostatic Zn plating, including the competition between Zn^2+^ reduction and parasitic reactions (HER/corrosion) that drive local pH change and generate interphase products; we do not intend to replicate sealed-cell constraints such as separator-confined mass transport, stack pressure or long-duration/high-capacity cycling, because the electron-transparent deposits must be formed directly on TEM grids for high-resolution TEM analysis. The sample preparation process was monitored by using an operando optical microscope to confirm the successful growth of zinc metal, as evidenced by a color change in the TEM film to a metallic gray (Fig. S1). The TEM grid with electroplated zinc was carefully disassembled from the electrochemical cell and gently rinsed with deionized water prior to further TEM analysis. Zinc electrodeposition was performed under three applied currents (10, 1 and 0.1 mA). At a fixed passed areal capacity, the higher-current condition produces a higher density of metal deposits and suppresses coarsening of the by-product (Fig. S2); accordingly, we adopt the highest-current condition (10 mA) in this work to most clearly resolve the nanoscale structure at the electrode–electrolyte interface.

**Figure 2.**
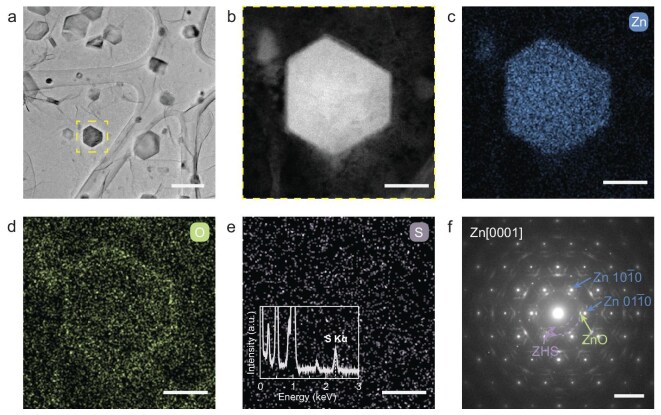
Identity verification of electroplated zinc complex under battery conditions. (a) Low-magnification TEM image of the electroplated sample. (b) High-angle annular dark-field scanning TEM (HAADF–STEM) image for the highlighted region in (a), with the corresponding energy-dispersive X-ray spectroscopy (EDS) elemental mappings of (c) zinc, (d) oxygen and (e) sulfur signal, along with the (f) selected-area electron diffraction (SAED) pattern. The inset in (e) shows the corresponding EDS spectrum for the sulfur signal. Scale bars are 200 nm in (a), 50 nm in (b–e) and 5 nm^−1^ in (f).

The identity of the prepared zinc samples was first confirmed by using TEM techniques. Low-magnification TEM imaging (Fig. 2a) revealed sparsely distributed and well-shaped crystals, indicating relatively uniform nucleation and the growth of highly crystalline zinc on the substrate. This morphology is representative of the initial charging stage in AZBs. A hexagonal crystal, as shown in Fig. 2a, was further characterized by using high-angle annular dark-field scanning TEM (HAADF–STEM, Fig. 2b). The hexagonal plate, with a side length of ∼120 nm, exhibited clear edges and corners, indicative of high crystallinity, despite the presence of a shell-like structure with a relatively lower contrast (see below). The elemental composition of the observed crystal was analysed by using energy-dispersive X-ray spectroscopy (EDS). The zinc elemental map (Fig. 2c) closely matched the hexagonal feature shown in Fig. 2b, confirming the zinc identity of the high-contrast hexagon. Meanwhile, the oxygen signal from the EDS map (Fig. 2d) revealed a shell-like structure surrounding the hexagon that was consistent with the lower-contrast shell observed in Fig. 2b, suggesting the presence of an oxide compound. The line profiles of the O signal in the high-resolution EDS mappings (Fig. S3) showed pronounced oxygen enrichment on the surface of the zinc crystal, indicating that ZnO is predominantly confined to the grain exterior shell, rather than penetrating into the bulk Zn grain. Additionally, a non-negligible sulfur signal (inset of Fig. 2e) was detected, although the elemental map (Fig. 2e) did not show a distinct spatial distribution.

To further characterize the observed features shown in Fig. 2b, selected-area electron diffraction (SAED) was performed (Fig. 2f). A group of sharp Bragg diffraction spots was observed in the SAED pattern, with two reflections representing their basal vectors pointed out by labeled arrows (Fig. 2f, *d* = 2.3 Å), which corresponds to the hexagonal close-packed (*hcp*) zinc along the [0001] orientation. Additionally, some slightly diffused satellite spots adjacent to the sharp zinc diffraction were identified (highlighted in green in Fig. 2f, *d* = 2.7 Å) and indexed to be the [0001] zone axis of wurtzite-structure ZnO with an epitaxial relationship with the hexagonal zinc plate. This confirms the existence of ZnO, which coincides with the O signal detected in the EDS (Fig. 2d). Furthermore, a diffraction ring with lattice spacing of 4.1 Å was distinguished, corresponding to the 110 reflection of the triclinic layered ZHS (Zn_4_(OH)_6_SO_4_·5H_2_O) (highlighted in purple, Fig. 2f) and consistent with the sulfur signals detected in the EDS analysis. A complete indexing of the SAED pattern for metallic zinc, ZnO and ZHS is provided in Note S1, along with their crystallographic structural models and simulated SAED patterns.

Although the time-resolved tracking of the same region from the initial Zn nucleation stage to a thick, continuous Zn film is technically challenging and beyond the scope of this study, we analysed a number of Zn deposits spanning a wide size range and consistently observed the same Zn–ZnO–ZHS hierarchical structure, suggesting that this SEI architecture is robust during Zn growth, particularly at the early plating stages (Note S2). While the formation of ZHS is frequently reported in works related to AZBs, the presence of ZnO in the mildly acidic electrolyte has been seldomly discussed [35]. To better understand the structural relationship between the zinc crystals and the shell-like ZnO, we further conducted a series of high-resolution STEM characterizations.

### The inner SEI: epitaxial ZnO coverall on metallic zinc

**Figure 3.**
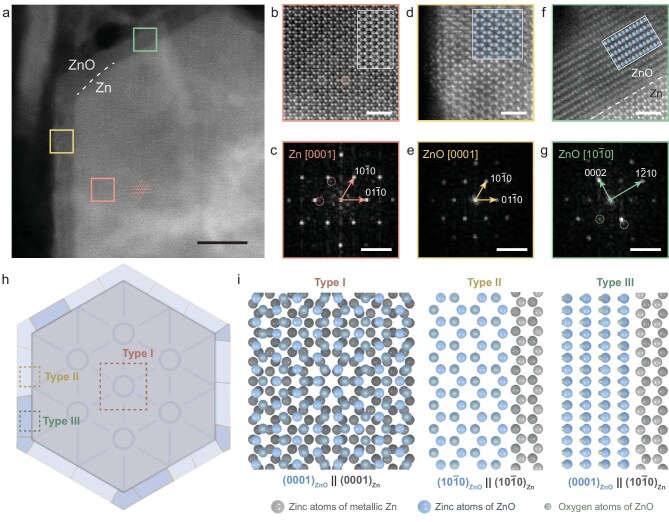
Epitaxial ZnO on the electroplated Zn metal. (a) High-angle annular dark-field scanning transmission electron microscopy (HAADF–STEM) image of a partial hexagonal-prism electroplated Zn covered by ZnO thin layer. (b, d and f) HAADF–STEM images of regions highlighted by pink, yellow and green boxes in (a), respectively, with their corresponding structure models overlapped as insets and their corresponding Fourier diffractograms (FDs) shown in (c, e and g). (h) Schematic illustration of the Zn–ZnO complex along the [0001] direction of the Zn. (i) Schematics of atomic structures for Type I, Type II and Type III Zn–ZnO complexes, corresponding to the pink, yellow and green boxes in previous panels. The pink dashed lines in (a) indicate the Moiré pattern from the Zn–ZnO interference. Pink circles in (b, c) highlight ZnO features, while green circles in (g) correspond to Zn features. Scale bars are 10 nm in (a), 1 nm in (b, d and f) and 5 nm^−1^ in (c, e and g).

Spherical-aberration-corrected (*C*_s_-corrected) HAADF–STEM was employed to characterize the zinc complex along the [0001] zone axis of the metallic zinc (Fig. 3a). A ∼4-nm-thin layer with significantly lower contrast compared with the metallic zinc was observed surrounding the hexagonal zinc crystal, consistently with the previously identified core–shell structure (Fig. 2b). A region from the (0001) plane of the zinc crystal (pink box in Fig. 3a) was further zoomed in on and denoised, as shown in Fig. 3b. Notably, atomic columns that do not belong to the expected structure of metallic zinc (pink circles in Fig. 3b) appear. Meanwhile, the corresponding Fourier diffractogram (FD) revealed additional peaks besides the expected patterns for zinc metal; these peaks can be attributed to the ZnO [0001] zone-axis pattern, which is epitaxially grown on the Zn (0001) surface (representative peaks of ZnO marked with pink circles with *d* = 2.8 Å, Fig. 3c). Owing to the epitaxial Zn–ZnO structure, the periodic contrast in the atomic arrangement forms a distinctive Moiré pattern, as shown in Fig. 3a (representative lattice marked with dashed pink lines), which is further confirmed in a high-resolution TEM image (Fig. S4). The Moiré lattice exhibited a spacing of 12.8 Å for a supercell *a*_m_ = *b*_m_ = 14.8 Å, *θ*_m_ = 60°, consistent with the interference between the lattice fringes of the zinc and ZnO as given by (*a*_ZnO_ × *a*_Zn_)/(*a*_ZnO_ − *a*_Zn_). Such an epitaxial overlapping of ZnO and Zn along their [0001] direction is defined as the Type I Zn–ZnO structure in this work.

Additionally, epitaxial ZnO was also observed attached to the {10$\overline{1}$0} surfaces of the metallic zinc crystal as polycrystalline grains. Two distinctive grains were studied (yellow and green boxes in Fig. 3a that are further presented in Fig. 3d and f, respectively). Figure 3d shows atomic columns corresponding to the projected structure of ZnO along its [0001] incidence, which is further confirmed by its FD (Fig. 3e). This epitaxially coated {10$\overline{1}$0} ZnO along the {10$\overline{1}$0} surface of the Zn, with their [0001] orientation aligned with each other, is denoted as Type II, despite having the same ZnO orientation as Type I. Interestingly, we also identified a [10$\overline{1}$0] ZnO grain with its (0001) surface attached to the Zn {10$\overline{1}$0} surface (Fig. 3f), supported by the peaks of ZnO from the FD corresponding to the 0002 and 12^¯^   10 reflections, with *d* spacings of 2.6 and 1.6 Å, respectively (Fig. 3g). Their epitaxial relationship can be determined by examining the relative positions of the peaks of the Zn metal from the FD (green dashed circles in Fig. 3g). This Zn–ZnO interfacial structure is designated as Type III Zn–ZnO in this work.

Based on these observations, we constructed models demonstrating the structure of ZnO on the {0001} or {10$\overline{1}$0} surface of the metallic zinc (Fig. 3h), where three types of epitaxial structure were defined (atomic structures in Fig. 3i):

Type I: ZnO (0001) on Zn (0001) with [2$\overline{11}$0]_ZnO_ ∥ [2$\overline{11}$0]_Zn_
Type II: ZnO (10$\overline{1}$0) on Zn (10$\overline{1}$0) with [0001]_ZnO_ ∥ [0001]_Zn_
Type III: ZnO (0001) on Zn (10$\overline{1}$0) with [10$\overline{1}$0]_ZnO_ ∥ [0001]_Zn_

These proposed structures were further distinguished and confirmed through HAADF–STEM by imaging the metallic zinc along its [2$\overline{11}$0] orientation (Fig. S5).

The observed thin layer of ZnO on the Zn metal may appear contradictory to the mildly acidic nature of the electrolyte used for the sample preparation. However, the formation of a ZnO layer on electroplated zinc metal is not uncommon, particularly in alkaline electrolytes [36–39], where it has been reported to originate from the interaction between zinc ions and hydroxide ions. It is important to note that the mild acidity of the electrolyte employed in this work—and more broadly in many AZBs—only represents the global pH of the bulk electrolyte and does not necessarily reflect the local pH near or on the electrode surface. Considering the inevitable occurrence of HER and Zn corrosion within and near the electrochemical double-layer region, the local generation and accumulation of hydroxide ions can significantly elevate the pH, creating a microenvironment that resembles that of alkaline electrolytes [40,41]. More importantly, AZBs are typically assembled under ambient conditions, in which both the aqueous electrolyte and the cell are exposed to air. Thus, any dissolved oxygen in the electrolyte—as well as gaseous oxygen from the atmosphere—can also contribute to the ZnO formation through spontaneous reactions with metallic zinc [42]. The detailed mechanistic pathway of ZnO formation, however, requires further operando characterizations [43]. In the context of battery applications, while the bulk ZnO exhibits semiconducting properties that may hinder the electrochemical process, the presence of a ZnO nanolayer on the electroplated zinc could be beneficial. It may facilitate the transport of zinc ions rather than hydrated ions and form a slightly passivating layer that resists further zinc corrosion by water [44–47].

### The outer ‘SEI’: ZHS flakes rooted from the edges of Zn crystals

Besides the zinc metals and the discovered ZnO nanolayer, greater interest may be drawn to the common by-product of ZHS in a ZnSO_4_-based electrolyte [35]. ZHS is generally composed of layered double hydroxides of brucite-like Zn(OH)_2_ layers containing SO_4_^2−^ anions and neutral H_2_O molecules positioned between the layered structure, making it extremely sensitive to external energy stimulation such as electron-beam irradiation or heating [48,49]. To preserve the ZHS flakes to the greatest extent in our experiments, we conducted a tolerance test of electron-beam irradiation on ZHS samples. The critical doses for ZHS under 300-keV electrons were determined by evaluating the intensity decay (to *e*^−1^ of its initial value, where *e* is the base of the natural logarithm) of the 410 diffraction rings of the ZHS and were calculated to be 105 and 207 *e*·Å^−2^ under ambient and liquid-nitrogen temperatures, respectively (see details in Note S3). To unravel the larger features of ZHS and its unique spatial relationship with Zn crystals while maintaining the metastable interfacial structure, we conducted cryoET to probe and reconstruct the spatial organization between the ZHS and the Zn crystals covered by the ZnO nanolayer.

CryoET is usually carried out on a cryogenic transmission electron microscope equipped with a direct-detection electron-counting camera that ensures optimal imaging performance under low-dose conditions. In this work, a typical workflow began with the systematic acquisition of tilt-series micrographs across an angular range of about ±60° (in total, 120° tilt) with 2° incremental steps (Fig. 1c); the tilt-series data were then reconstructed to form a 3D volumetric dataset—referred to as a tomogram—through numerical algorithms, which was further segmented based on deep-learning-based approaches to distinguish various features (see details in the Supplementary data).

**Figure 4.**
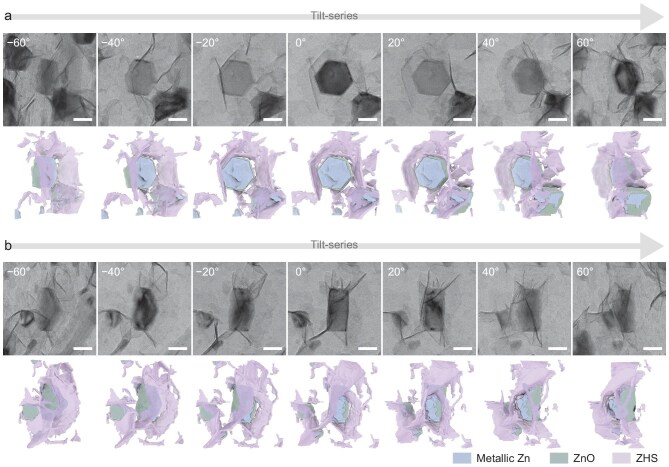
3D visualization of hierarchical solid–electrolyte interphase (SEI) structure for electroplated zinc. Tilt-series TEM images and their reconstructed 3D models for the Zn–ZnO–ZHS complex oriented to the (a) [0001] and (b) [2$\overline{11}$0] directions of the metallic zinc. The reconstructed 3D models were rotated to the same tilting angle as the representative projection images for visualization purposes. Zn, ZnO and ZHS are distinguished by blue, green and purple in the reconstructed 3D model, respectively. Scale bars are 100 nm.

Two typical orientations of the electroplated zinc complex were selected for cryoET characterization to obtain a detailed spatial relationship between the Zn, ZnO and ZHS. A zinc complex oriented along the [0001] direction of the metallic Zn crystal was first examined (Fig. 4a and Video S1), where the central part appeared as a hexagonal Zn crystal with a lateral dimension of ∼125 nm and a thickness of ∼70 nm (hexagonal prism in the middle of the reconstructed 3D model shown in Fig. 4a). Consistent with prior observations, the reconstructed model revealed full coverage by a thin ZnO layer (4.6∼5.1 nm) on the Zn surface (green features in the reconstructed 3D model shown in Fig. 4a), although ZnO was not visible on the top facet of the Zn crystal due to limitations in data collection, known as the ‘missing wedge’ [50]. Furthermore, the ZHS flakes were successfully segmented from the zinc complex (purple features in the reconstructed 3D model shown in Fig. 4a), showing typical side lengths of 70∼180 nm and thicknesses of 6.1∼6.6 nm. Interestingly, a unique spatial relationship between the Zn–ZnO particles and the ZHS flakes was identified. The ZHS flakes primarily originated from the edges of the Zn–ZnO hexagonal prism and extended outward into the bulk electrolyte region, forming a petal-like configuration at an angle of 117°∼160° relative to the {0001} surface of the metallic Zn (see Supplementary data for details). This petal-like arrangement of ZHS flakes at the Zn–ZnO edges was further confirmed in a truncated hexagonal prism-shaped Zn–ZnO particle oriented along the [2$\overline{11}$0] direction of the metallic zinc (Fig. 4b and Video S2). Using the reconstructed 3D model, the microvolume of each component in the Zn–ZnO–ZHS complex was semi-quantitatively estimated. The ZnO and ZHS components were found to consume 19∼29 wt% of the zinc content in the entire zinc complex (Fig. S6), clearly illustrating the significant loss of Zn mass efficiency during the initial stage of the battery charging process.

**Figure 5.**
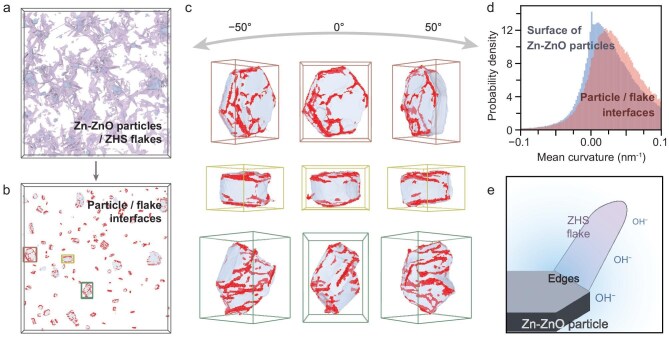
Analysis of the interface between Zn–ZnO particles and ZHS flakes. (a) Reconstructed 3D model for electroplated zinc complex, where Zn–ZnO particles and ZHS flakes are in blue and purple, respectively. The box is 1.629 μm × 1.629 μm × 0.208 μm in size. (b) Zn–ZnO particles from (a), where their interfaces with ZHS flakes are highlighted in red. (c) Representative Zn–ZnO particles highlighted in (b) at different viewing angles. (d) Probability density distribution of mean curvature for the surface of the Zn–ZnO particles and Zn–ZnO particle/ZHS flakes interfaces. (e) Schematic illustration of the chemical gradient of hydroxide ions near the edge of the Zn–ZnO particle.

To further investigate the unique edge effect of the ZHS configuration, a detailed analysis of the interface between the ZHS flakes and the Zn–ZnO particles was conducted on >100 zinc crystals (Fig. 5a and Video S3). Based on the reconstructed models, the interfaces between the Zn–ZnO particles and the ZHS flakes were identified and are visualized in Fig. 5b, in which the highligted regions indicate the contact boundaries of the ZHS flakes with the Zn–ZnO particles. Notably, these red-marked interfaces highlight a strong edge-related characteristic regardless of the dimensions of the Zn–ZnO particles or their orientations with respect to the substrate. In particular, three individual Zn–ZnO particles with their ZHS interfaces highlighted are shown in Fig. 5c, further demonstrating the strong spatial association of the ZHS flakes with the geometric edges of the Zn–ZnO particles, along with a possible correlation with the step features present on the crystal faces of the Zn–ZnO particles. Furthermore, we evaluated the mean curvature of the Zn–ZnO surfaces and the interfaces between the Zn–ZnO particles and the ZHS flakes, and summarized the results as a probability density distribution of the mean curvature (Fig. 5d). Notably, the curvature corresponding to the highest probability density for the Zn–Zn particle/ZHS flake interfaces lies at ∼0.01 nm^−1^, intersecting with that of the Zn–ZnO particles at 0.024 nm^−1^. The distinct shift toward positive curvature for the interfaces compared with the Zn–ZnO surface, accompanied by a broader tail at higher curvature values, indicates that the ZHS flakes appear preferentially on the convex, highly curved regions of the Zn–ZnO surface, which corresponds to the edge feature of the Zn–ZnO particles and further supports the edge-philic growth of the ZHS flakes on the Zn–ZnO particles.

Typically, in AZBs utilizing a ZnSO_4_ electrolyte, the formation of ZHS flakes results from the local accumulation of OH^−^ ions, which are primarily generated by the competing HER during the battery charging process [51]. As the ZHS flakes are observed to preferentially originate from the edges of the Zn–ZnO particles and extend outward, this suggests a likely local elevation of the concentration of hydroxide ions and a preferential nucleation site at the edges of the Zn–ZnO particles (Fig. 5e). Based on this observation, we speculate that the edges of the Zn–ZnO particles may exhibit higher activity toward processes generating OH^−^, such as HER, compared with the basal planes, thereby leading to the preferential formation of ZHS at these edge sites besides the possible preferential nucleation. This hypothesis is consistent with recent first-principles studies showing that the HER kinetics on Zn are sensitive to surface atomic coordination and facet identity [52]. Additionally, this tendency could also arise from several non-exclusive factors, including (i) the ‘tip effect’ in which protrusions/high-curvature regions concentrate the local electric field and favor localized reactions [53], (ii) Zn self-dissolution/corrosion that can locally raise the pH and promote by-product formation [54], and (iii) during ZHS precipitation, the heterogeneous nucleation preferentially occurs at crystal imperfections such as grain boundaries, edges and corners [55]. The outward extension of the ZHS flakes at an angle of (142 ± 16)° (*n* = 32) relative to the {0001} surface of the Zn may result from an optimal supply of necessary ions—including OH^−^, sulfate ions (SO_4_^2−^) and zinc ions (Zn^2+^)—enabled by the minimal physical barriers to ion transport presented by the basal plane and vertical facets of the Zn–ZnO particles, while some potential interfacial configurations between the Zn–ZnO and the ZHS may also contribute. Interestingly, this speculation regarding the chemical activity of the Zn-crystal edges aligns with previous observations, which demonstrated improved battery performance when using textured Zn (0002) surfaces that expose fewer edges and exhibit a higher coulombic efficiency with a longer cycling life [56–58].

## CONCLUSIONS

In this study, we investigated the detailed structure of electroplated zinc and its SEI under battery-relevant conditions by using TEM and cryoET techniques. The electroplated zinc exhibited a hierarchical interfacial structure, consisting of an inner SEI layer of ZnO and an outer SEI structure of ZHS flakes. The ZnO nanolayer is epitaxially coated on the electroplated Zn metals, forming three defined crystallographic relationships: (0001)_ZnO_ ∥ (0001)_Zn_, (10$\overline{1}$0)_ZnO_ ∥ (10$\overline{1}$0)_Zn_ and (0001)_ZnO_ ∥ (10$\overline{1}$0)_Zn_; meanwhile, the ZHS flakes were found to originate preferentially from the edges of the Zn–ZnO particles, forming a petal-like configuration. This unique composition and spatial arrangement of the extended SEI highlight the local chemical heterogeneity that arises during battery operation, such as a potential accumulation of hydroxide ions at the Zn-crystal edges induced by HER or Zn corrosion. The integration of TEM and cryoET reveals this atomic-scale heterogeneity and multidimensional architecture of subtle interphases, providing a comprehensive understanding of the underlying interfacial structure of zinc negative electrodes. These findings shed light on the heterogeneous chemical environment within and near the electrochemical double-layer region during battery charging, offering fundamental insights for the future designs of AZBs, particularly in zinc texture design.

## Supplementary Material

nwag114_Supplemental_Files
