# Supplementary material for: Three-dimensional and nanoscale resolved hierarchical structure of electroplated zinc complex in aqueous zinc battery
Source: Natl Sci Rev. 2026 Apr 13;13(9):nwag114. doi: 10.1093/nsr/nwag114 (PMC13182254; doi:10.1093/nsr/nwag114)
Supplement: nwag114_Supplemental_Files [file nwag114_supplemental_files.zip › Supplementary data.pdf]

Supplementary data for

Three-dimensional and nanoscale resolved hierarchical structure of electroplated zinc complex in aqueous zinc battery

Junyan Li[1,†], Xun Guan[1,†], Jing Wang[1], Ge Zhang[1], John Holoubek[1], Yi Cui[1], Yuqi Li[1], Haoya Wang[1], Wah Chiu[2,3,4] and Yi Cui[1,5,6,*]

[1]Department of Materials Science and Engineering, Stanford University, Stanford, CA 94305, USA;

[2]Department of Bioengineering, Stanford University, Stanford, CA 94305, USA;

[3]Department of Microbiology and Immunology, Stanford University School of Medicine, Stanford, CA 94305, USA;

[4]Division of CryoEM and Bioimaging, SSRL, SLAC National Accelerator Laboratory, Menlo Park, CA 94025, USA;

[5]Stanford Institute for Materials and Energy Sciences, SLAC National Accelerator Laboratory, Menlo Park, CA 94025, USA;

[6]Department of Energy Science and Engineering, Stanford University, Stanford, CA 94305, USA

***Corresponding author**. E-mail: yicui@stanford.edu

[†]Equally contributed to this work.

Table of Contents

Methods

Supplementary Notes 1–4

Supplementary Figures 1–15

Supplementary Videos 1–3

Supplementary References

Methods

Materials and chemicals

Zinc sulfate heptahydrate salt ($ZnSO_4 \cdot 7H_2O$, Sigma-Aldrich 31665), zinc foil (Thermo Fisher 010436), and TEM grids with single-layer graphene (Ted Pella Inc. 21710) were used as received. All deionized (DI) water was obtained from a Millipore water purification system.

Sample preparation

The zinc deposition was conducted in a homemade two-electrode system, which was fabricated on an acrylic sheet by laser cutting (Epilog Fusion M2 Laser Cutter at Stanford Nano Shared Facilities). The working electrode was a single-layer graphene TEM (transmission electron microscopy) grid connected by a copper tape as the lead; the counter electrode was a piece of freshly cut zinc foil with an exposed geometric area of ~ 7 mm^2, comparable to the geometric area of the TEM grid. The two electrodes were placed side by side with a distance of 5 mm to ensure the crystal growth on the TEM grid's film. Prior to sample deposition, ~ 1 mL of 2 M $ZnSO_4$ electrolyte was added to the cell, which ensures the coverage of both the working and the counter electrode; then the cell was covered by a thin optical glass, which also flattened the electrolyte in the electrochemical cell. A constant current of −10 mA was applied to the working electrode by a Biologic VMP3 system for a typical duration of 2 s, the cell voltage was monitored meanwhile. *Operando* optical observation of the working electrode was conducted by an Olympus BX51 microscope at 10× magnification equipped with a Swiftcam SC1803.

TEM characterization

TEM and scanning TEM (STEM) experiments were performed on a Thermo Fisher Spectra Double-Corrected Transmission Electron Microscope (Thermo Fisher Scientific Inc.) operated at 300 kV. Before imaging, the spherical aberration corrector was aligned using a standard gold sample. The selected zinc crystal was first tilted to the corresponding zone axes for selected-area electron diffraction (SAED) pattern acquisition under parallel-beam conditions. Then, the microscope was switched to STEM mode for further imaging or spectroscopy studies. High-resolution TEM (HRTEM) image was collected on an FEI Titan Environmental Transmission Electron Microscope equipped with an image spherical aberration corrector. For critical dose

evaluation of zinc hydroxide sulfate (ZHS) under cryogenic temperature (at the accelerating voltage of 300 kV), the specimen was first loaded in a Gatan ELSA 698 cryo-transfer holder and cooled to liquid nitrogen temperature after being inserted into the microscope. TEM or STEM data were processed using the Gatan DigitalMicrograph software or the *hyperspy* Python package[1]. The denoised filter used for high-angle annular dark-field STEM (HAADF-STEM) images is a nonlinear filter from the *hrtem_filter* python package[2, 3]. SAED patterns were simulated using SingleCrystal software.

Cryogenic electron tomography (cryoET) data acquisition

CryoET experiments were carried out on a Thermo Scientific Glacios cryo-transmission electron microscope (Thermo Fisher Scientific Inc.) equipped with an X-FEG Schottky-emission electron gun (operated under an acceleration voltage of 200 kV) and a Cryo-Autoloader. Bright field TEM images were acquired with two different fields of view of 1.63 μm and 0.496 μm by a Falcon 4i (4096 × 4096 pixels in total, Thermo Fisher Scientific Inc.) direct electron detector, corresponding to the high-magnification (Fig. 4) and low-magnification (Fig. 5) data, respectively. An objective aperture of 70 μm was used during imaging. To control the electron-beam irradiation, data were collected under low electron dose conditions — 1.4 $e{\cdot}Å^{-2}$ and 13.6 $e{\cdot}Å^{-2}$ per frame for low magnification imaging and high magnification imaging, respectively. Image series with tilting range from −64° to 64° (tilt step 2°, 65 frames in total) were collected using the Tomography 5 software (Thermo Fisher Scientific Inc.). The focus value was determined and then set to −1 μm before every image acquisition. The focusing procedure was performed on an area outside the acquisition area by an image shift to mitigate electron-beam damage. Although electron irradiation damage under parallel-beam conditions may induce slight morphological changes to the beam-sensitive ZHS during the cryoET experiments, the observed morphology after irradiation remains largely consistent with that before exposure. Such evolution is negligible for the tomographic reconstruction and our subsequent analysis (see Supplementary Note 3 for details).

Tomography data processing

Reconstruction

Electron tomography tilt series were reconstructed into tomograms using IMOD[4]. Raw tilt images were first pre-processed with the default X-ray replacement to suppress sporadic high-

intensity artifacts, followed by a cross-correlation coarse alignment. We then performed fine alignment using the patch tracking method. In this step, IMOD automatically generated an array of 8 × 8 partially overlapping patches (patch size: 680 × 680 pixels). For convenient downstream editing, the parameter "break contours into pieces" was set to 7. IMOD subsequently tracked the patch trajectories across the entire tilt series; the resulting patch contours were inspected manually, and contour segments exhibiting severe drift were removed to reduce the alignment residual error. After alignment, tomogram positioning was performed with default settings to determine the reconstruction thickness and refine the tilt geometry. The final aligned stack (with a binning factor of 4) was reconstructed to a tomogram by a weighted back-projection algorithm, and an IMOD 20-run simultaneous iterative reconstruction technique (SIRT)-like filter was applied to the reconstruction.

Segmentation

The reconstructed tomograms were subsequently imported into Dragonfly 2024.1 software (Comet Technologies Canada Inc., Montreal, Canada; software available at https://www.theobjects.com/dragonfly) for segmentation. Differentiating the signal from zinc crystals or ZHS in the TEM images solely based on their relative intensity through thresholding is not feasible, as their thickness varies significantly over a broad range, and the intensity is affected by diffraction contrast, phase contrast, and artifacts from tomography reconstruction (Supplementary Note. 4). To address this issue, several sections from the tomogram were arbitrarily selected for manual labeling of zinc crystals, zinc oxide, ZHS, and background based on the morphologies observed in the TEM images through the following five steps: (i) The Zn metal phase, which is typically characterized by a clear geometric morphology and well-defined, straight/regular edges in tomogram sections, were firstly annotated. This phase was delineated using the Brush/Polygon tool in Dragonfly. For the sections closed to a horizontal (top or bottom) surface where the edge may be blurred due to the "missing wedge" effect, the edges of zinc crystal could be determined by the contrast difference across several adjacent sections. (ii) We then annotated the ZnO nanolayer as the relatively weaker-contrast region immediately outside the Zn boundary, forming a thin conformal shell consistent with our independent STEM imaging evidence of an epitaxial ZnO interlayer reported earlier in the manuscript. Due to the limited resolution, zinc oxide was not segmented in the low-magnification datasets. (iii) Next, we labeled the ZHS phase

as the external filamentous/strip-like (petal-like) features extending outward from the interface. (iv) All remaining unlabeled voxels in these sections were assigned as background. (v) After annotating multiple representative interior sections, we additionally annotated one section near the top (or bottom) of the tomogram and explicitly labeled the reconstruction-artifact-dominated regions as background, because tomographic reconstruction may introduce artifacts (e.g., missing-wedge elongation, streaking, and boundary-related artifacts near the top/bottom of the reconstructed volume). This step helps prevent the network from learning artifact-associated features as any material phase. Typically, for the high-resolution tomogram (Fig. 4a), manual annotation was performed on 5 sections out of a total of 291 sections (~1/60 of 1024 × 1024 × 291 voxels for the whole dataset). For the low-magnification tomogram (Fig. 5), in total, 2 sections (8 × 1/4 section) out of 131 sections were manually annotated, corresponding to a similar annotation fraction. The annotated sections were used for a supervised training with a U-Net architecture implemented in Dragonfly's deep-learning module. After convergence, the trained network was applied to the entire tomogram to generate the full 3D segmentation. Furthermore, by iteratively correcting predictions and retraining, model accuracy would further be improved. The same model could be applied to additional datasets with the same pixel size and intensity calibration, demonstrating both the efficiency and generalizability of the segmentation workflow. After segmentation, individual zinc particles were separated by 3D connected-component labeling using a 26-connectivity criterion on the segmented zinc volumetric data. The intersecting ZHS network was split by a watershed algorithm. The segmentation results were visualized using UCSF ChimeraX software[5].

Curvature analysis

Mean curvature calculation was carried out in Dragonfly software. Specifically, the Zn-ZnO particles segmented from the tomography data were first subjected to a "ROI Dilate" operation with a kernel size of 3. The resulting three-dimensional Zn geometry was then converted into a triangular mesh, which was subsequently smoothed (5 iterations). Finally, the mean curvature of all mesh faces can be computed, and the faces in contact with the ZHS volume can be determined through the "Compute Measurements" module.

Tomographic morphometry

The semi-quantitative chemical distribution (Fig. S6) for each reconstructed Zn complex in Fig. 4 was evaluated using the volume from the tomography data and the crystallographic densities of metallic zinc, zinc oxide, and ZHS.

Morphologic characteristics, such as the dimensions of zinc metal and ZHS flakes, were measured directly in the segmented model, as shown in Fig. S7a–d and i–l. Volume distribution profiles as a function of model height were calculated by summing the voxels assigned to Zn, ZnO, or ZHS in each horizontal layer of the segmented model (Fig. S7e and m). ZnO was consistently observed in conjunction with the Zn core, while ZHS flakes extended from the substrate and distributed beyond the top surface of the Zn-ZnO structure, forming an outer layer with a total thickness of ~ 140 nm. The out-of-plane thicknesses of ZnO and ZHS were estimated using local thickness mapping (Fig. S7g–h and o–p, respectively). Most of the ZnO layers exhibit a thickness of 4.6 ~ 5.1 nm, whereas the thickness of ZHS flakes is 6.1 ~ 6.6 nm. The angles between individual ZHS flakes and the adjacent Zn crystals, measured on the cross-sections from tomograms, are 117 ~ 160° (Fig. S8).

The absence of ZnO on the top surface of the Zn crystal in Fig. 4 (see isolated models of ZnO in Fig. S7c and k) can be attributed to the missing wedge effect — a known limitation in electron tomography arising from incomplete angular sampling at high-tilt angle, which can obscure top and bottom surfaces in reconstructions. Nevertheless, the presence of a ZnO layer on the top surface has been previously confirmed by the observation of Moiré fringes in spherical aberration-corrected STEM imaging.

Supplementary Notes

Supplementary Note 1: Structures and phase identification against selected area electron diffraction (SAED)

An electroplated zinc sample on a graphene substrate is composed of metallic Zn, ZnO, and zinc hydroxide sulfate (ZHS). Their structural models and crystallographic information are given in Fig. S9a–c, respectively. Wurtzite-structure ZnO can grow epitaxially on Zn along its [0001] orientation. We simulated their SAED patterns along the [0001] direction, as shown in Fig. S9d and e. The overlapped SAED pattern of [0001] Zn and ZnO (Supplementary Fig. 9f) matches well with the experimental SAED pattern in Supplementary Fig. 9g, confirming the epitaxial relationship between the metallic Zn crystal and ZnO coverall.

The diffuse scattering features of ZnO can be explained by a combined effect of (i) mosaicity in the ZnO nanolayer and (ii) double diffraction arising from the coherent overlap of Zn and ZnO domains along the beam direction[6].

To support this interpretation, we performed simplified simulations that intentionally focus on the key physical origins of the observed patterns (Fig. S10). Specifically, rather than using atomistic models, we treated Zn and ZnO as ideal unit-cell-based reciprocal lattices and separately simulated: (1) the double-diffraction contribution expected from overlapping Zn and ZnO domains—convolution of their reciprocal-lattice spot sets (Fig. S10a), and (2) the mosaicity-induced diffuse scattering of ZnO by applying an orientation distribution (mosaic spread) that converts discrete reflections into diffuse features (Fig. S10b). We then convolved these two components to generate the composite diffraction pattern (Fig. S10c).

Notably, the resulting simulated pattern reproduces the main qualitative characteristics of the experimental data, including the coexistence of sharp reflections and diffuse intensity, and the overall spot distribution and symmetry, showing good agreement with Fig. 2f. These results support our conclusion that the diffuse scattering is primarily associated with ZnO mosaicity disorder, while additional spots/intensity arise from Zn/ZnO double diffraction under coherent overlap.

In order to index the diffraction rings consist of weak diffraction spots in the experimental pattern. We calculated the one-dimensional intensity profile by rotational average over the pattern,

depicted in Fig. S9g. The intensity profile and the corresponding background fitting using a power function were plotted in Fig. S9h, where the weak diffraction signals are well resolved. Five peaks, in addition to Zn and ZnO, have been distinguished. In a zoomed-in profile (Fig. S9i), four of them could be indexed to 100, 110, 210, and 410 of ZHS ($Zn_4(OH)_6SO_4 \cdot 5H_2O$). Notably, ZHS is regarded as a series of basic sulfate salts with similar intralayer structure, but different amounts of interlayer water, as depicted in Fig. S9c. These reflections ($l = 0$) present in the SAED pattern can also be assigned to other ZHS variants that share the same intralayer structure.

Meanwhile, a diffraction signal corresponding to graphene $10\bar{1}0$ was observed. Consequently, the experimental diffraction pattern has confirmed the growth of zinc, ZnO, and ZHS complex on the graphene substrate.

Supplementary Note 2: Evidence for interphase persistence during Zn growth

Transmission electron microscopy (TEM) images in Fig. S11 show a broad range of Zn crystallite sizes (~30–237 nm) from 11 distinct zinc particles, which consistently reveal the same hierarchical Zn-ZnO-ZHS motif. Across these growth states, that is, different particle sizes, the epitaxial ZnO layer remains thin (~3–6 nm) and does not show pronounced thickening with increasing Zn size, suggesting that the ZnO interphase forms rapidly at the early stage and is thereafter relatively stable in thickness (within our sampling and resolution). In contrast, 3D volumetric analysis based on a lower-magnification tomogram (Fig. S12) indicates that ZHS accumulation evolves with Zn growth: ZHS volume increases approximately linearly with Zn crystallite size at the initial stage but slows for larger crystallites, consistent with progressively stronger local ion depletion and/or mass-transport limitations in the developing 3D architecture. Together, these semi-quantitative results support that the hierarchical Zn-ZnO-ZHS interphase is established early and persists as a general structural motif during Zn growth, while ZHS evolves in a size-dependent, transport-influenced manner.

Supplementary Note 3: Evaluation of electron-beam damage for zinc hydroxide sulfate (ZHS)

ZHS possesses a binary structure with a dense layer constructed by strong bonds and interstitial water molecules connected via hydrogen bonds (Fig. S9c), which makes the ZHS highly sensitive under electron-beam irradiation. Indeed, diffraction rings in SAED for ZHS rapidly attenuate and get diffused when the 300 keV electron was induced, indicating destructive damage to the crystallographic structure of ZHS (Supplementary Figs. 13a–c). Meanwhile, morphological shrinkage on the edge of ZHS flakes was also observed (Supplementary Figs. 13d–f). Quantitative analysis on the SAED patterns shows a rapid intensity decay of reflections 210 and 410 at room temperature (Supplementary Figs. 14a and c), while the other sharp peaks correspond to graphene remaining intact during electron-beam irradiation. The critical dose (accumulated dose that the diffraction signal reduces by a factor e^{-1}, e is the base of the natural logarithm) for ZHS in the sample, calculated from the intensity of reflection 410, is 105 $e{\cdot}Å^{-2}$ (Fig. S14e).

By cooling the specimen to liquid N_2 temperature, cryogenic transmission electron microscopy (cryoTEM) can stabilize the metastable interface. ZHS shows better beam irradiation tolerance for 300 keV electrons under cryogenic conditions (Supplementary Figs. 14b and d). The critical electron dose of ZHS increases to approximately 207 $e{\cdot}Å^{-2}$ (Fig. S14e).

Electron doses were carefully controlled during our cryogenic electron tomography (cryoET) experiments. For low-magnification datasets (Fig. 5), only 91 $e{\cdot}Å^{-2}$ per tilt series was applied. However, substantially higher doses — up to 884 $e{\cdot}Å^{-2}$ per dataset — were applied during high-magnification data acquisitions to ensure a sufficient signal-to-noise ratio for tomographic reconstruction. Notably, tomography performed on a 200 kV microscope would result in more severe radiolysis compared to a 300 kV one, which potentially leads to greater beam-induced damage[7]. Nevertheless, the morphology of ZHS only changes slightly following electron beam irradiation (Fig. S13). More importantly, it is well established that the process of three-dimensional (3D) reconstruction from projection images inherently averages out uncorrelated noise and artifacts present in individual images[8]. Given the negligible beam-induced morphological alterations observed and the fact that our study focuses primarily on overall morphological features rather than atomic-resolution structures, the reconstruction results obtained via cryoET remain valid for providing reliable 3D representations of ZHS morphology.

Supplementary Note 4: Intensity non-uniformity in tomogram and its impact on segmentation

Electron tomography data acquired under parallel-beam TEM conditions can exhibit contrast variations caused by contrast transfer function (CTF) modulation and diffraction contrast. Although STEM (e.g., HAADF-STEM) tomography can mitigate diffraction and phase-contrast artifacts, it typically requires a substantially higher electron dose compared to TEM tomography, which is not well-suited for our system because the electrolyte-derived interphase is unstable under electron irradiation.

Because Zn is highly crystalline, diffraction contrast changes markedly across the tilt series. In our dataset, this is evident in representative tilt images: the hexagonal Zn crystal at the center appears noticeably darker in Fig. S15a than in Fig. S15b. These diffraction-contrast variations, together with the intrinsic phase contrast in TEM imaging, propagate into the reconstruction and manifest as non-uniform intensity within a single Zn particle in the tomogram (representative tomogram section are shown in Fig. S15d). Consequently, conventional intensity-based thresholding becomes unreliable, and simple contrast thresholding fails to produce meaningful segmentations. Fig. S15e–g explicitly illustrates this failure mode under conditions dominated by diffraction and phase contrast.

To verify that the defocus condition we used in the cryoET experiments does not compromise the morphology of deposited Zn or the subsequent tomography segmentation, we performed a lowpass filtering test on a representative high-magnification projection from our tomography dataset. The resulting lowpass-filtered image (Fig. S15c) preserves all morphological features of the Zn deposit, indicating that the information required for morphology interpretation and segmentation is contained predominantly in the low-frequency band below the first CTF zero.

Importantly, although the Zn crystal shows internal intensity non-uniformity, the interfacial boundaries remain sharp and well defined in the tomogram. The three constituent components—Zn metal, the epitaxial ZnO nanolayer, and ZHS flakes—can therefore be distinguished robustly based on their morphology and spatial arrangement. Together with our independent HAADF-STEM characterization of the Zn–ZnO interface presented earlier in the manuscript, we consider manual identification and annotation of these phases to be reliable (Fig. S15h). Moreover, the clear and consistent contour features make this dataset well-suited for deep-learning-assisted

segmentation, which is inherently less sensitive to absolute intensity variations than global thresholding.

Supplementary Figures

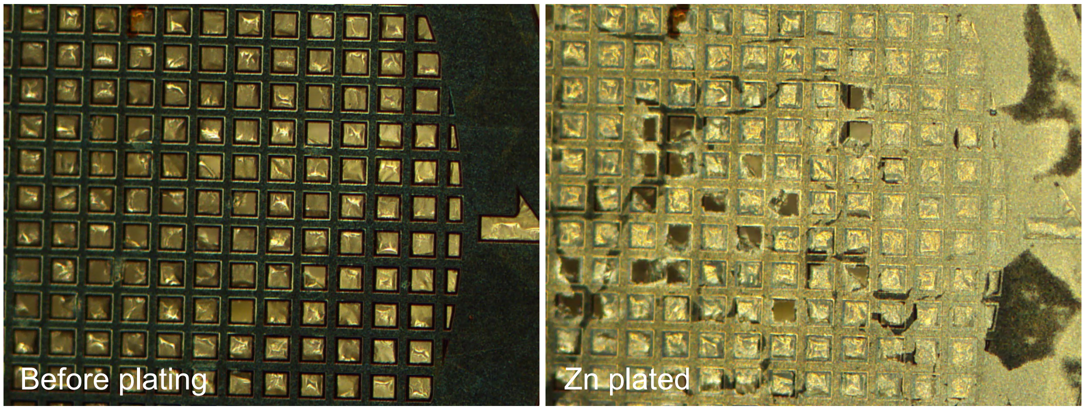

Supplementary Figure 1. Typical optical dark-field images of the TEM grid before and after zinc electroplating.

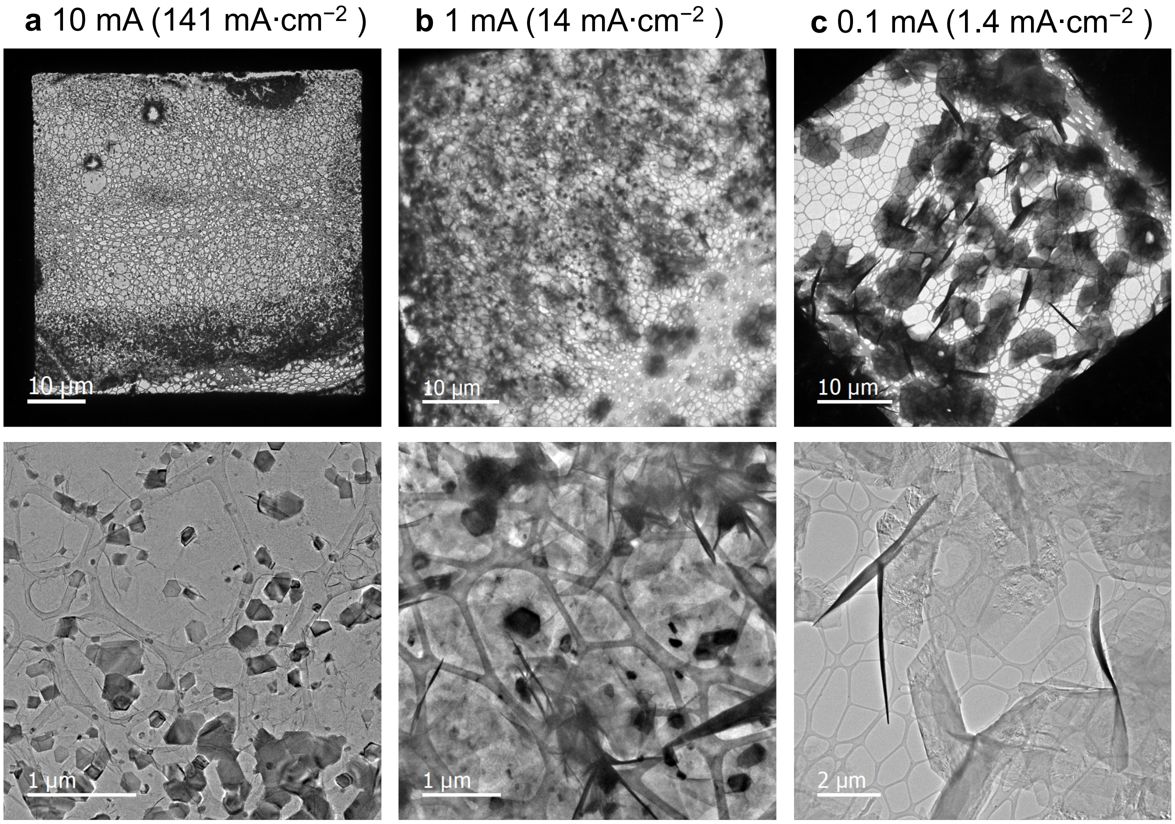

Supplementary Figure 2. Current-density-dependent Zn deposition and interphase formation on graphene TEM grids at identical areal capacity. Representative low- (top) and high-magnification (bottom) TEM images of Zn electrodeposits prepared at (a) 10 mA (141 mA·cm^{-2}), (b) 1 mA (14 mA·cm^{-2}), and (c) 0.1 mA (1.4 mA·cm^{-2}). The passed areal capacity was kept constant at 0.0786 mAh·cm^{-2} by adjusting deposition time to 2 s, 20 s, and 200 s, respectively.

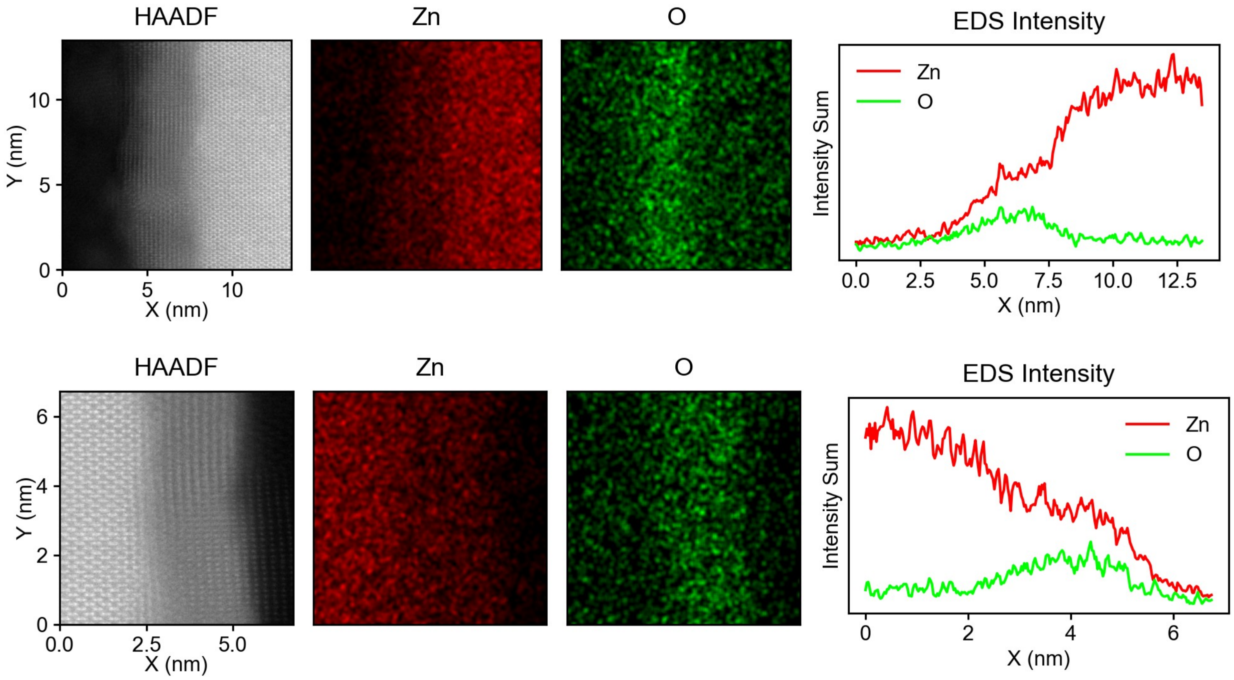

Supplementary Figure 3. Edge-localized oxygen distribution at the Zn grain surface revealed by scanning transmission electron microscopy (STEM) energy dispersive X-ray spectroscopy (EDS). Representative atomic-resolution high-angle annular dark field (HAADF)-STEM images (left) with corresponding EDS elemental maps of Zn (red) and O (green), and EDS intensity profiles integrated along the Y axis (right) collected across the Zn grain boundary/surface region for two representative areas (top and bottom rows). In both cases, the oxygen signal is strongly enriched near the grain periphery while the Zn signal increases toward the grain interior, indicating that oxygen-containing species are primarily confined to the surface region, consistent with a ZnO shell-like interphase rather than bulk oxygen penetration.

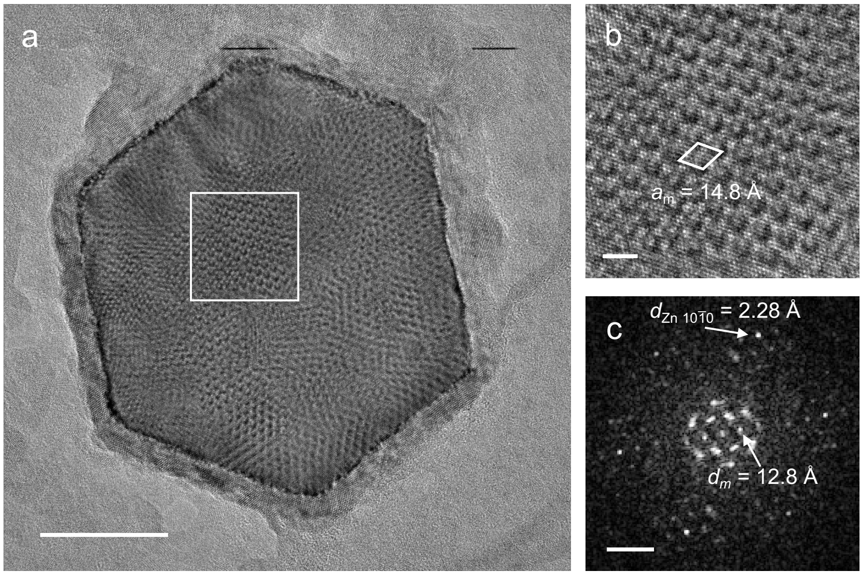

Supplementary Figure 4. Moiré pattern on the electroplated zinc crystal. (a) HRTEM image taken along the metallic zinc's [0001] incidence. (b) Region of interest highlighted with a white box in (a). The Moiré lattice was measured to be: $a_m = b_m = 14.8$ Å, $\theta_m = 60°$. (c) Fourier diffractogram (FD) of (b). Scale bars are 20 nm in (a), 2 nm in (b), and 2 nm^{-1} in (c).

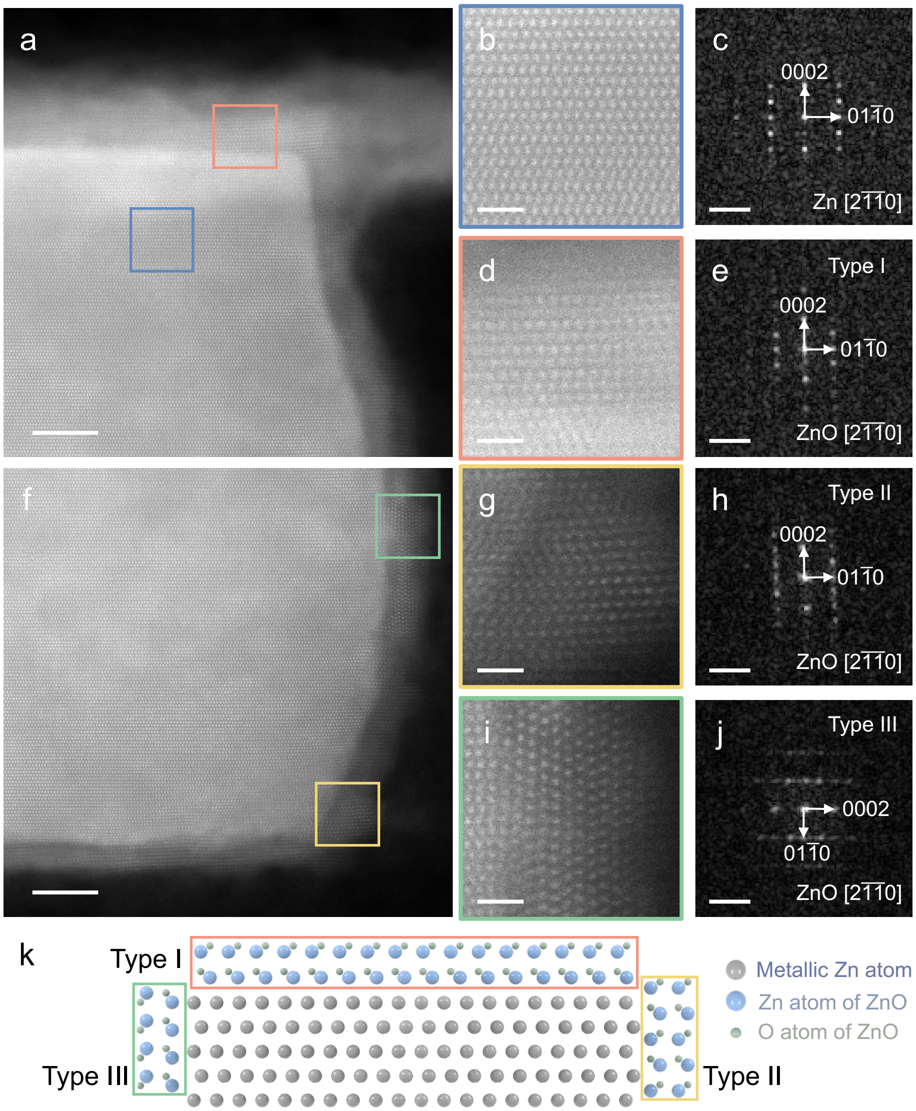

Supplementary Figure 5. High-angle annular dark-field scanning transmission electron microscopy (HAADF-STEM) micrographs of electroplated zinc complex along metallic zinc's [2$\bar{1}\bar{1}$0] incidence. (a and f) HADDF-STEM micrographs of a Zn-ZnO particle with regions of interest. (b, d, g, and i) Magnified images of the blue, red, yellow, and green highlighted regions in (a and f), respectively, with the corresponding FDs in (c, e, h, and j). (k) Schematics of the atomic structure for the epitaxial ZnO on metallic zinc. Scale bars are 5 nm in (a) and (f), 1 nm in (b, d, g, i), and 5 nm^{-1} in (c, e, h, j).

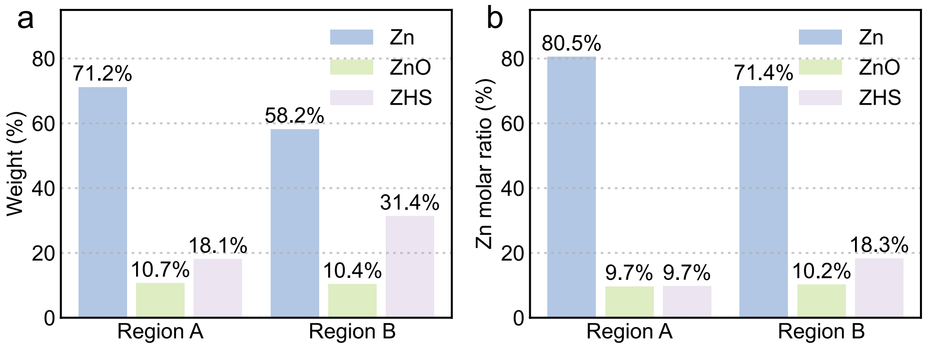

Supplementary Figure 6. Semi-quantitative evaluation of Zn, ZnO, and zinc hydroxide sulfate (ZHS) components in the electroplated zinc complex. Mass ratio (a) for zinc metal, ZnO, ZHS, and molar ratio (b) of zinc element in these three components calculated from Fig. 4a (region A) and Fig. 4b (region B), respectively.

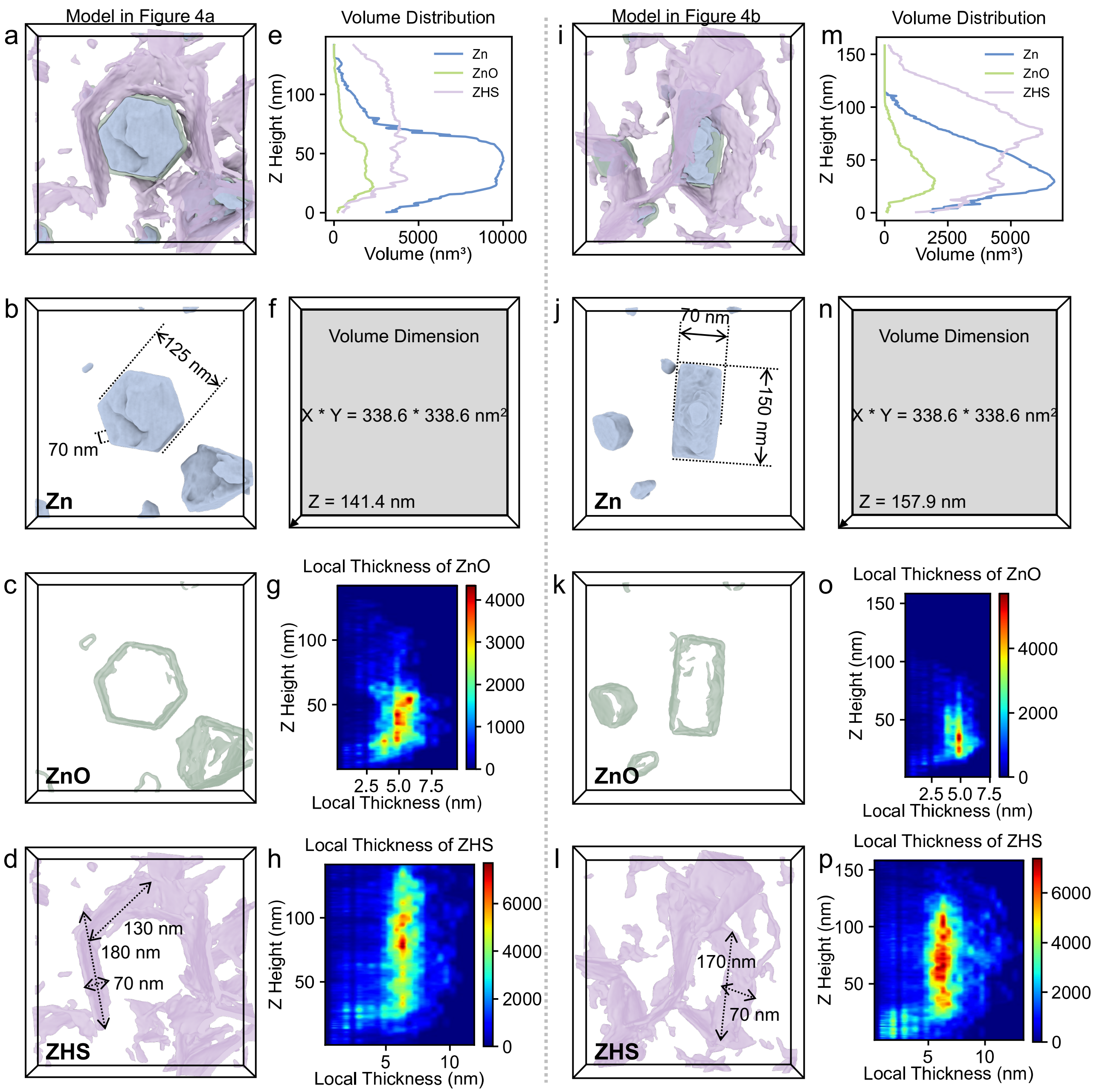

Supplementary Figure 7. Semi-quantitative morphological analysis for electroplated zinc. 3D visualizations of reconstructed models from tomographic data for (a and i) the whole zinc complex, (b and j) the metallic zinc, (c and k) the ZnO coverall, (d and i) and the zinc hydroxide sulfate (ZHS) flakes with their (e and m) the volume distribution statistics, (f and n) the dimensions of the 3D volume, and the local thickness of (g and o) ZnO and (h and p) ZHS. Particles in (a to h) and (i to p) correspond to those in Fig. 4a and b along metallic zinc's [0001] and [$2\overline{1}\overline{1}0$] directions, respectively.

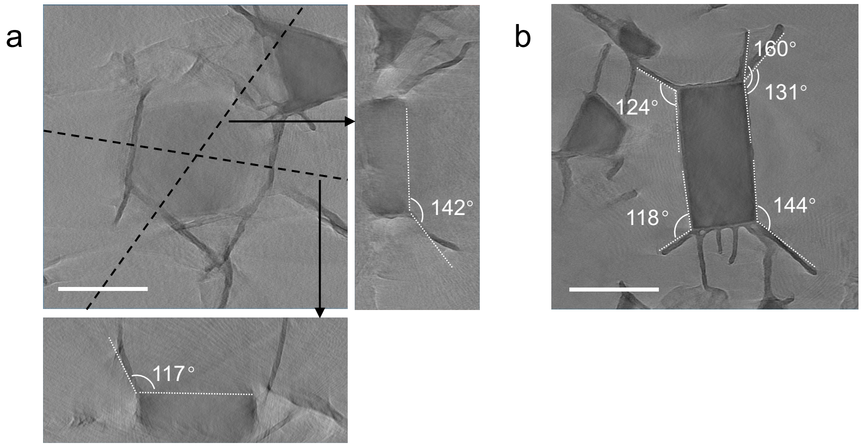

Supplementary Figure 8. Angle measurements for the constructed tomograms. Connecting angle between zinc hydroxide sulfate (ZHS) flakes and adjacent {0001} plane of metallic zinc crystal for the two electroplated zinc complexes oriented along (a) zinc [0001] and (b) $[2\bar{1}\bar{1}0]$ directions, respectively. Slice positions of side-view sections in (a) shown on the left and the bottom are pointed out by dashed lines in the overlook section. Scale bars are 100 nm.

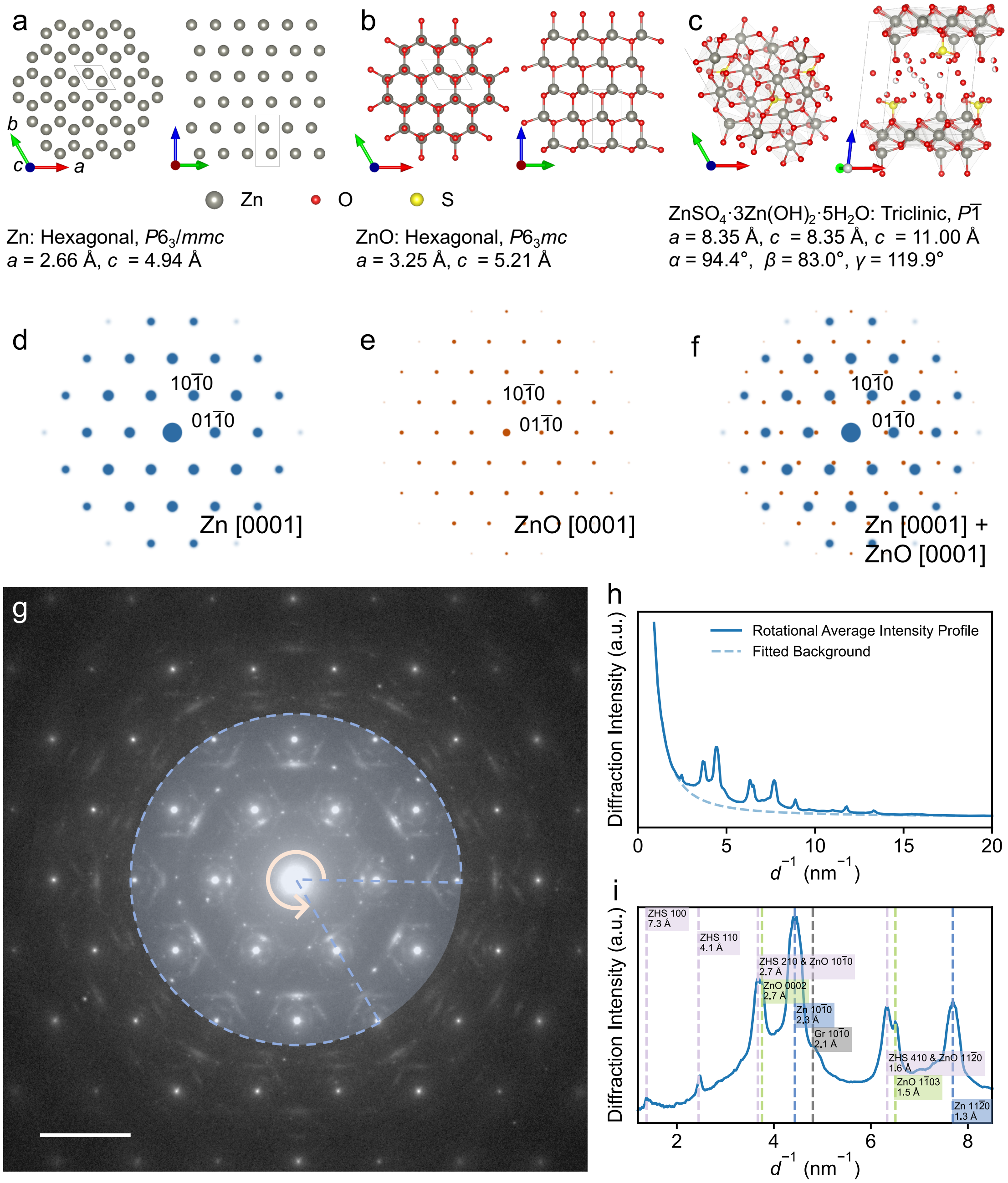

Supplementary Figure 9. Indexing of the selected-area electron diffraction (SAED) pattern of electroplated zinc complex on graphene substrate. (a–c), Structural models of (a) zinc, (b) zinc oxide, and (c) zinc hydroxide sulfate (ZHS) visualized using Vesta software[9]. (d and e) Simulated SAED patterns of zinc and zinc oxide along their [0001] orientation, respectively. (f) Overlap of diffraction pattern in (d and f). (g) Schematic of rotational average of SAED pattern corresponds to Fig. 2f in the main text. Scale bar is 5 nm^{-1}. (h) Rotation-average intensity profile and its background fitting for the diffraction pattern in (g). (i) Indexing of the diffraction pattern.

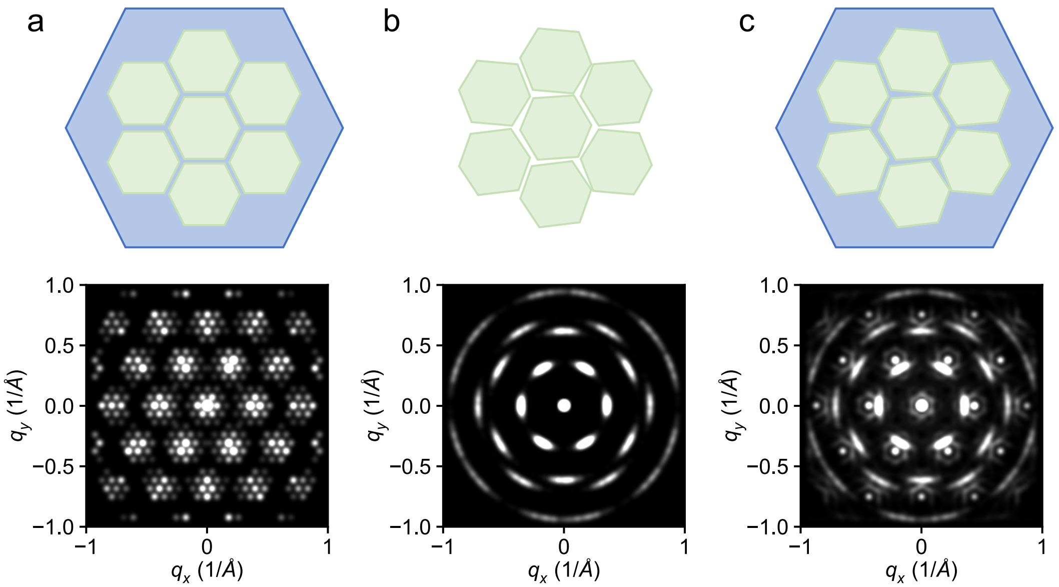

Supplementary Figure 10. Schematic models (top) and simplified diffraction simulations (bottom) illustrating the origin of complex diffraction for epitaxial intergrowth Zn-ZnO along [0001] orientation. (a) Model for coherent overlap of Zn and ZnO domains along the beam direction, generating double diffraction effect. (b) Model representing the mosaic ZnO, producing ring-like diffuse intensity. (c) Combined model incorporating ZnO mosaicity (orientation spread) together with the Zn-ZnO double-diffraction contribution; the resulting simulated pattern reproduces the coexistence of sharp reflections and diffused scattering observed experimentally.

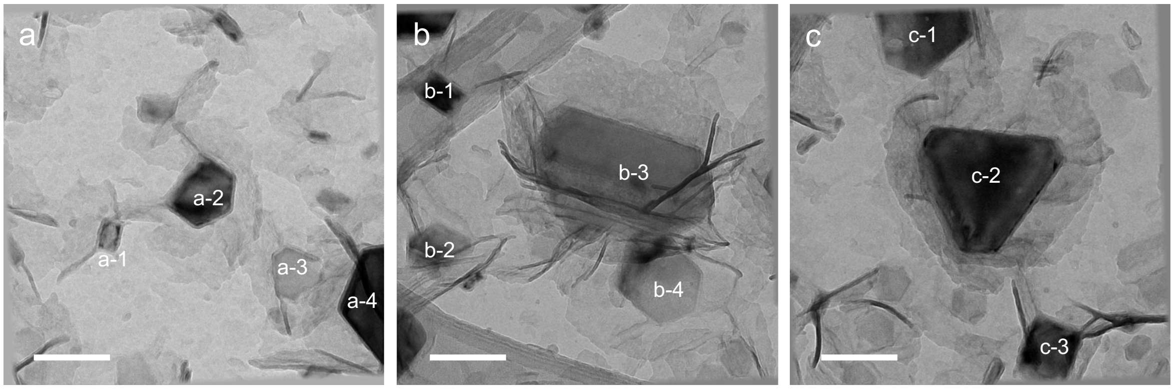

Supplementary Figure 11. TEM images of hierarchical Zn-ZnO-zinc hydroxide sulfate (ZHS) structure with different zinc sizes. The labeled zinc particles were used for semi-quantitative analysis of structural evolution. Scale bars are 100 nm.

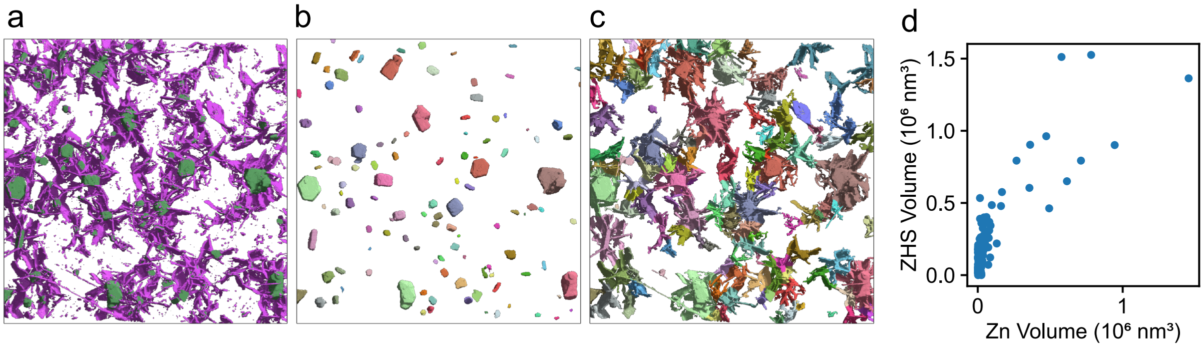

Supplementary Figure 12. 3D segmentation and volumetric correlation of Zn and zinc hydroxide sulfate (ZHS) within a representative tomogram sub-volume (1.6 μm × 1.6 μm). (a) Segmented Zn-ZnO core (green) and ZHS (purple) from tomogram. (b) 3D connected-component rendering of individual Zn-ZnO particles within the same sub-volume, with each particle colored uniquely for particle-wise quantification. (c) 3D rendering of the separated Zn-ZnO-ZHS complex in the same sub-volume, with individual ZHS flakes/fragments colored uniquely. (d) Scatter plot of ZHS volume versus Zn volume for the paired components quantified in this sub-volume, illustrating the particle-to-particle variability and the tendency.

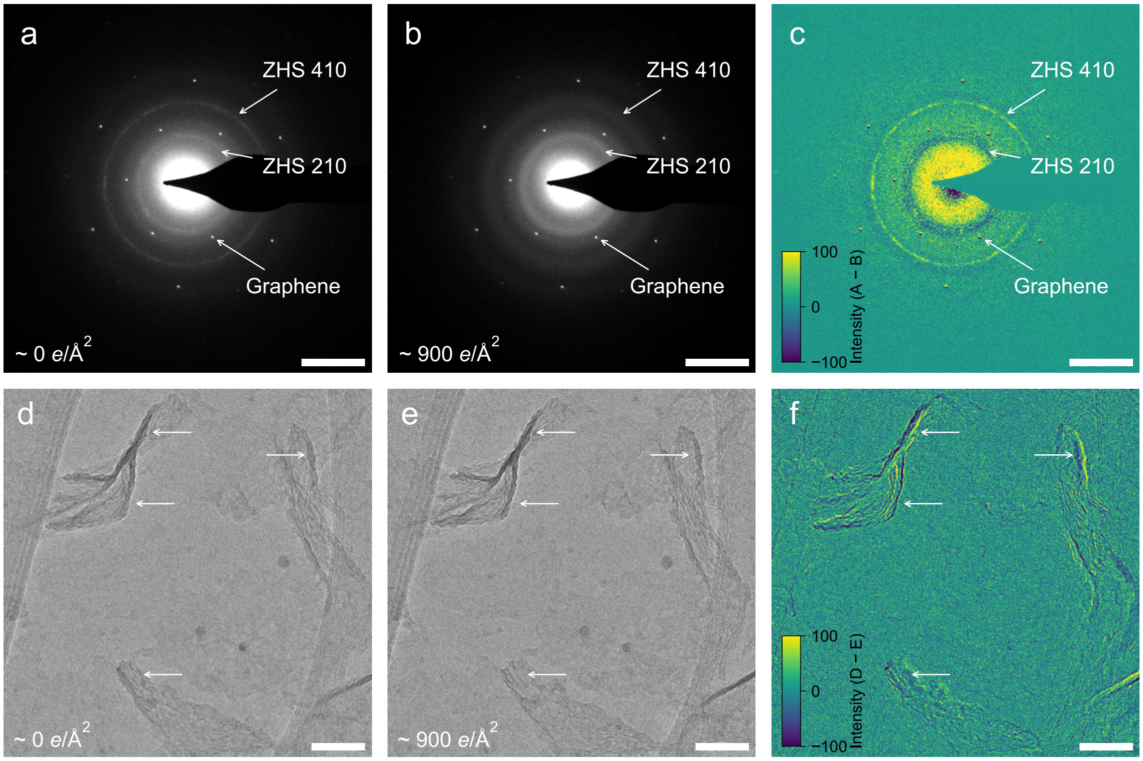

Supplementary Figure 13. Diffraction and morphology evolution of zinc hydroxide sulfate (ZHS) under 300 keV electron irradiation at room temperature. (a–c) SAED patterns and (d–f) TEM images of ZHS before (a and d) and after (b and e) electron-beam damage, and (c and f) the intensity difference for the corresponding images or diffraction patterns. Deformations of ZHS flakes were pointed out by white arrows in TEM images (d–f). Scale bars are 5 nm^{-1} in (a–c) and 50 nm in (d–f).

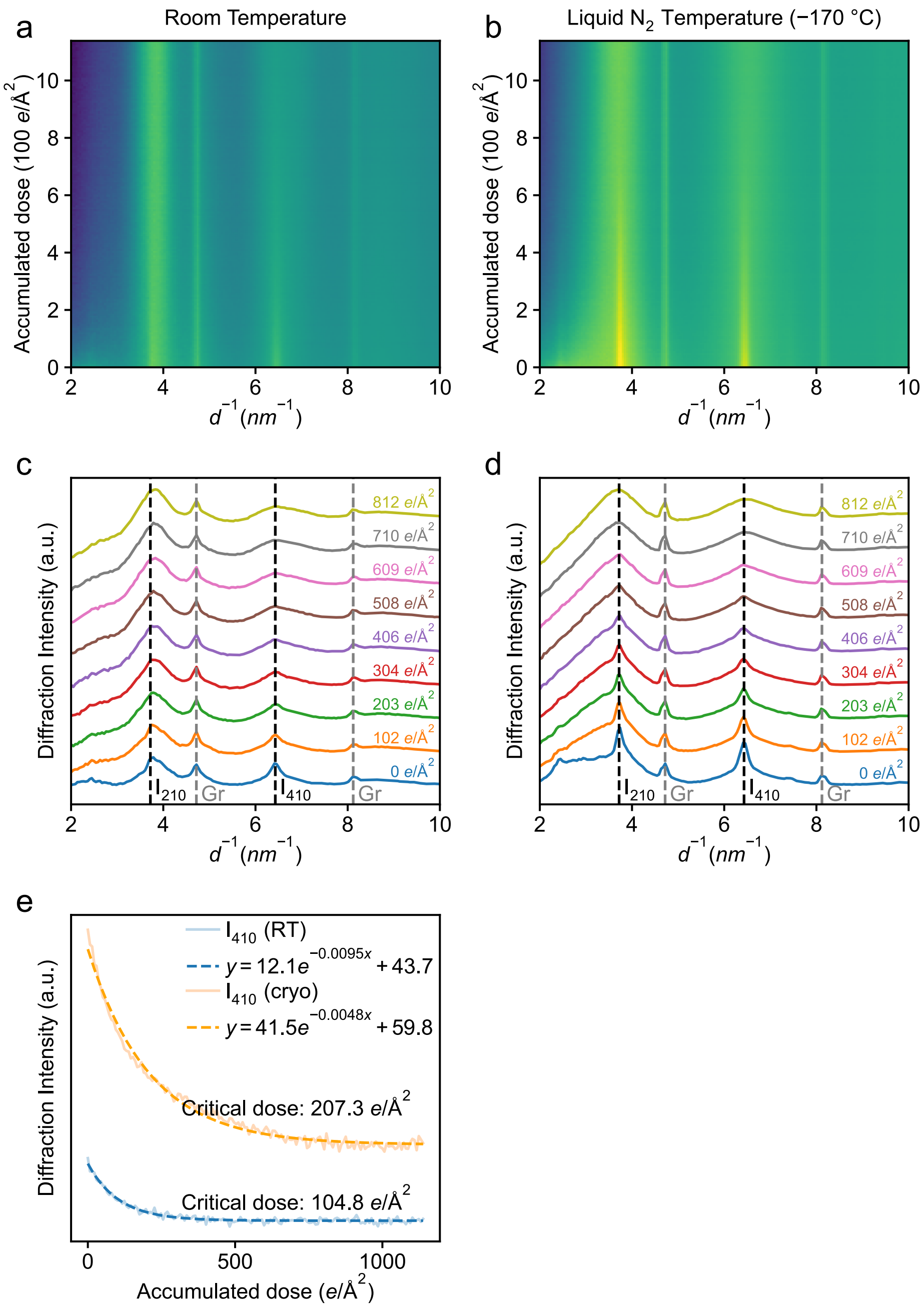

Supplementary Figure 14. Electron-beam irradiation damage of zinc hydroxide sulfate (ZHS) at room temperature and cryogenic temperature. Electron-dose-dependent diffraction intensity for ZHS under 300 keV electron irradiation at (a) room temperature and (b) cryogenic temperature. (c and d) Diffraction intensity profiles extracted from (a) and (b), respectively, for different electron doses. The vertical dashed lines indicate the reflections 210, 410 of ZHS, and the reflections belong to graphene, respectively. (e) Exponential fitting of the intensity attenuation for reflection 410 as a function of accumulated dose, indicating that the cryogenic condition has increased the critical dose of ZHS by twice.

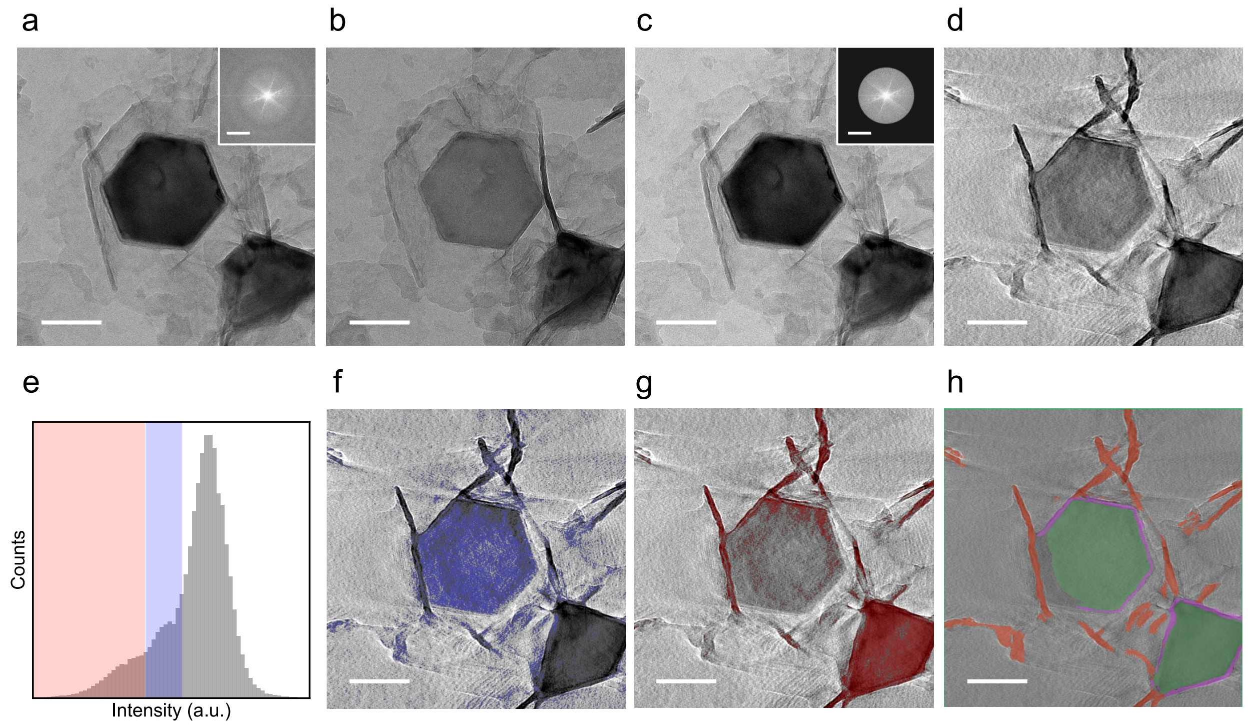

Supplementary Figure 15. Segmentation comparison between intensity thresholding and deep learning algorithm. (a and b) Representative raw tilt-series transmission electron microscopy (TEM) images acquired under parallel-beam conditions showing strong diffraction-contrast variations within crystalline Zn particles. (c) Lowpass-filtered image by zeroing spatial frequencies higher than 0.65 nm^{-1} in the Fourier space of image a (corresponding to the first zero crossing of the TEM contrast transfer function). (d) Slice extracted from the corresponding reconstructed tomogram, where these contrast modulations persist and appear as non-uniform intensity inside individual Zn grains. (e) Grayscale histogram of tomogram section in (d), pixels with intensity in the two selected ranges (red and blue) were highlighted in (f and g). (f and g) Results of simple intensity-threshold segmentation (blue/red overlays) demonstrating the failure mode: incomplete/fragmented Zn regions and spurious classification of surrounding features due to diffraction-contrast intensity fluctuations. (h) Manual morphology-/geometry-guided annotation of the three constituent phases—Zn metal (green), epitaxial ZnO nanolayer (purple), and zinc hydroxide sulfate (ZHS) flakes (orange)—enabled by clearly defined interfacial contours, despite internal intensity non-uniformity. Scale bars in (a–d) and (f–h) are 100 nm. Insets in (a and c) show the corresponding Fourier diffractograms with their scale bars of 0.5 nm^{-1}.

Supplementary References

1. Peña Fdl, Prestat E, Lähnemann J *et al.* Hyperspy/hyperspy: V2.3.0: Zenodo; 2025.
2. Du H. A nonlinear filtering algorithm for denoising HR(S)TEM micrographs. *Ultramicroscopy* 2015; **151**: 62–7.
3. Ma T. Python implementation of various denoising filters for HR(S)TEM images. *Microsc Microanal* 2024; **30**: 457–9.
4. Kremer JR, Mastronarde DN, McIntosh JR. Computer visualization of three-dimensional image data using imod. *J Struct Biol* 1996; **116**: 71–6.
5. Pettersen EF, Goddard TD, Huang CC *et al.* UCSF ChimeraX: Structure visualization for researchers, educators, and developers. *Protein Sci* 2021; **30**: 70–82.
6. Edington JW. Electron diffraction in the electron microscope. *Electron diffraction in the electron microscope*. London: Macmillan Education UK; 1975. 1-77.
7. Egerton RF. Mechanisms of radiation damage in beam-sensitive specimens, for TEM accelerating voltages between 10 and 300 kV. *Microsc Res Tech* 2012; **75**: 1550-6.
8. Kak AC, Slaney M. *Principles of computerized tomographic imaging*: Society for Industrial and Applied Mathematics, 2001.
9. Momma K, Izumi F. VESTA 3 for three-dimensional visualization of crystal, volumetric and morphology data. *J Appl Crystallogr* 2011; **44**: 1272-6.
