# Supplementary material for: Three-dimensional and nanoscale resolved hierarchical structure of electroplated zinc complex in aqueous zinc battery
Source: Natl Sci Rev. 2026 Apr 13;13(9):nwag114. doi: 10.1093/nsr/nwag114 (PMC13182254; doi:10.1093/nsr/nwag114)
Supplement: nwag114_Supplemental_Files [file nwag114_supplemental_files.zip › Teaser text.docx]

The three-dimensional nanoscale architecture of electroplated zinc is resolved by advanced TEM and cryoET, revealing the spatially heterogeneous electrochemical reactivity and informing the design of high-performance aqueous zinc batteries.
